# Agreement study on gait assessment using a video-assisted rating method in patients with idiopathic normal-pressure hydrocephalus

**DOI:** 10.1371/journal.pone.0224202

**Published:** 2019-10-24

**Authors:** Masatsune Ishikawa, Shigeki Yamada, Kazuo Yamamoto

**Affiliations:** 1 Rakuwa Villa Ilios, Kyoto, Japan; 2 Normal Pressure Hydrocephalus Center, Rakuwakai Otowa Hospital, Kyoto, Japan; 3 Department of Neurosurgery, Rakuwakai Otowa Hospital, Kyoto, Japan; Goethe University Hospital Frankfurt, GERMANY

## Abstract

Gait disturbance is a major symptom of idiopathic normal-pressure hydrocephalus (iNPH) and is assessed by raters of different professions or with different degrees of experience. Agreement studies are usually done by two raters or more, and comparisons among multiple groups of raters are rare. In this study, we aimed to examine the agreement among multiple groups of raters on gait patterns and a grading scale through a video-assisted gait analysis in patients with iNPH. Fifteen participants with iNPH were enrolled. Gait was assessed according to seven patterns, including freezing and wide-based gaits. The levels of severity (evident, mild, none) were rated by three groups of raters (two neurosurgeons [DR2], three experienced physiotherapists [PTe3], and two less experienced physiotherapists [PTl2]) through a simultaneous video viewing session. Severity of gait disturbance (GSg) was rated using the Japanese iNPH grading scaleiNPHGS, and Krippendorff alpha was computed to assess agreement, with alpha ≥0.667 indicating good agreement and alpha ≥0.8 indicating excellent agreement. For group comparisons, 84%, not 95%, confidence intervals were applied. Among the seven gait patterns in the first assessment, excellent agreement was observed in wide-based and short-stepped gaits in only DR2. Good agreement was observed in four patterns, but the agreement by two groups was in shuffling and wide-based gait. There were no gait patterns showing good agreement among three groups. In the second assessment, excellent agreement was observed in three patterns but no gait patterns showed good agreement between two groups or more. Learning effect was observed only for standing difficulty in DR2. In contrast, good or nearly good agreement on GSg was observed among the three groups with excellent agreement in two groups. Agreement on gait patterns among the three groups of raters was not high, but agreement on the iNPHGS was high, indicating the importance of a precise description facilitating differentiation between neighboring grades.

## Introduction

Gait disturbance is common in various disorders among elderly people. Short-stepped, shuffling, and freezing gait patterns with turning difficulty are reported to be major features of gait disturbance in idiopathic normal-pressure hydrocephalus (iNPH) [1,2]. Of note, such gait disturbance is similar to that in Parkinson disease, but Stolze et al. [3] reported that the specific features of gait disturbance in iNPH were a wide-based gait with outward-rotated feet and a shuffling gait.

Gait disturbance is the most frequent symptom of iNPH [1]. Marmarou et al. [4] reported several degrees of gait disturbance: high (difficulty in tandem walking in 93% and imbalance in 85% of patients), moderate (difficulty in turning in 48%, wide-based gait in 43%, and difficulty in rising from a chair in 33% of patients), and low (magnetic gait in 17% of patients). Souza et al. [5] reported gait disturbances in velocity, cadence, step height, and step length as well as *en bloc* turning and *en bloc* gait in approximately 80% of patients. However, these percentages concerning patterns of gait disturbance may vary among multiple raters.

For gait analysis, computerized three-dimensional gait analyses are useful for the quantitative analysis of gait disturbance [6], but such analyses are often complex and expensive to perform. The recent advent of smartphones and their applications allows quantitative measurement of each compartment on the Timed Up and Go Test (TUG) [7, 8]. [The upgraded version in English, “Hakaro iTUG,” is downloadable from Apple’s iPhone App Store^®^.] However, observational gait analysis (with or without video) is a popular method of gait analysis and is useful for data collection. Agreement studies are usually done by two raters or more [9], but comparisons among multiple groups of raters are rare [10].

In the present study, agreement among multiple groups of raters, from different professions and differing degrees of clinical experience with iNPH, was examined by observing videos of the gait disturbance during TUG in patients with iNPH and assessing the presence and severity of their gait patterns. The severity of their gait disturbance was also assessed using the gait domain (GSg) of the iNPH grading scale (iNPHGS) developed by Kubo et al. [11]. In addition, the effect of video-assisted learning session was investigated.

## Methods

### Participants

The study design and protocol were approved by the ethics committee for human research of Rakuwakai Otowa Hospital (Raku-Oto-Rin-17-010). The participants were recruited from March 2017 to October 2018. The participants or their representatives provided their written informed consent for study participation. All 15 participants had one or more of iNPH triad with magnetic resonance imaging (MRI) findings of ventriculomegaly, tight high convexity, and dilated Sylvian fissure (disproportionately enlarged subarachnoid space hydrocephalus; DESH) [1]. They responded well to the removal of 30-mL of cerebrospinal fluid via a lumbar tap (tap test) and showed improvements in their symptoms after undergoing ventriculoperitoneal shunting. As a result, they were all regarded as having definite iNPH [1].

### Gradings and video viewing

In the present study, video recordings of the TUG Test before the tap test were used to assess gait disturbance in iNPH. The TUG measures time for completion of six components: after receiving the go signal, the participants arise from the chair, walk straight to 3 m, turn around from a mark, walk back, turn back again, and finally sit down. It contains major components of gait in the daily life. Seven gait patterns were assessed: freezing gait, shuffling gait, wide-based gait, short-stepped gait, festination of gait, standing difficulty, and turning difficulty. Grades were assessed according to the following three categories: grade 0 (none), grade 1 (mild), and grade 2 (evident). In addition, the gait domain of the iNPHGS (GSg) was graded using a 0–4 scale: grade 0 (normal), grade 1 (complaints of instability), grade 2 (walks without supportive devices), grade 3 (walks with supportive devices), and grade 4 (unable to walk).

Video viewing was repeated twice in the first assessment session. Thereafter, a 15-minute video-assisted learning session of typical types of gait disturbance in iNPH with discussions was held in order to achieve consensus. During these sessions, the seven types of gait disturbance were defined as follows: freezing gait was a brief interruption of forward progression even though the feet seemed to move with hesitation (“start hesitation”); shuffling gait showed an individual whose feet appeared to be glued to the ground (“magnetic feet”); wide-based gait was characterized by a base wider than the normal; short-stepped gait showed a reduced stride length; festination was characterized by involuntary acceleration when walking; difficulty in standing and turning was assessed based on instability while standing and turning during the TUG. Severity of gait disturbance was also rated based on the GSg. After the learning session, a second assessment session was held.

### Raters

The seven raters who participated in this study were divided into three groups: two neurosurgeons (DR2), three experienced physiotherapists (PTe3), and two less experienced physiotherapists (<1 year of experience each; PTl2). Furthermore, the following two groups were added: all five physiotherapists (PTa5) and all seven participants (two neurosurgeons and five physiotherapists; All7). The DR2 group included two neurosurgeons who were specialized in the diagnosis and treatment of iNPH with 30 and 6 years of experience, respectively. The PTe3 group included experienced physiotherapists who had been assessing iNPH for >4 years each. The PTl2 group included less experienced physiotherapists with less than 1 year of experience each. Thus, the DR2, PTe3, and PTl2 groups were regarded as the homogeneous group. The mixed groups included the PTa5 and All7 groups.

### Data analyses

All statistical analyses were performed using the open source software program R version 3.3.2 (R Foundation for Statistical Computing, Vienna, Austria; http://www.R-project.org). Continuous data are presented as mean and standard deviation, and categorical data as prevalence rate and percentage. Statistical significance was set at 0.05 (two-tailed). Krippendorff alpha coefficients for agreement were computed by using the “rel” packages (version 1.3.1) of the R software program [12]. Krippendorff alpha coefficients allow for the rating of ordinal data and were used with multiple raters [13]. According to Krippendorff, alpha ≥0.8 is regarded as excellent and alpha ≥0.667 as acceptable. In the present study, alpha ≥0.667 was regarded as indicating good agreement. The confidence intervals (CIs) of the alpha coefficient were computed with bootstrapping, with repetitions set at 1,000 times. There are few available statistical methods that are designed to qualify the degree of agreement between two independent groups of raters—rather than two individual raters—on an ordinal scale [10]. To address this issue, 84% CIs, not 95% CIs, were computed based on the findings of MacGregor-Fors and Payton [14], who reported that 84% CIs robustly mimicked statistical tests at p<0.05. For comparison between two groups, overlapping of 95% CIs between two groups indicates no statistical difference. Non-overlapping of 95% CIs indicates statistically significant differences at p<0.01. When 84% CIs are applied to group comparison, non-overlapping of the 84% CIs suggests statistical difference at p<0.05, which is commonly used for statistical significance. For this reason, we apply 84% CIs in this study. Thus, the non-overlap of 84% CIs is regarded as indicating statistical significance at p<0.05. Pairwise comparisons between the first and second assessments and among the independent groups of PTe3, DR2, and PTl2 for each pattern of gait disturbance were performed using this approach. Variability of data was a prerequisite for chance-corrected agreement studies including the Krippendorff statistics. Lack of variation yields alpha = 0, which is meaningless for agreement study. In such cases, data were excluded from subsequent analyses [15].

## Results

The clinical summary of participants is shown in Table 1. Eight men and seven women participated in this study (mean age ±standard deviation: 77 ± 5.5 years). Falling (9/15: 60%) was the most frequent comorbidity, followed by dementia (6/15: 40%) and lumbar canal stenosis (4/15: 26.7%). The frequencies of grades in the modified Rankin scale (mRS) and the three domains of the iNPHGS in each participant are shown in Table 1. The mean cerebrospinal fluid pressure during the tap test was 13.1± 5.4 cmH_2_O. All participants were considered responders during the tap test and shunt surgery.

**Table 1:** Clinical summary of participants.

Case	Sex	Age (years)	Fall	AD	PD	CVD	LCS	mRS	GSg	GSc	GSu	Pcsf	Tap test	Shunt
**1**	**F**	**83**	**1**	**1**	**0**	**0**	**0**	**4**	**3**	**3**	**3**	**14**	**positive**	**VP**
**2**	**M**	**69**	**1**	**0**	**0**	**0**	**0**	**2**	**2**	**2**	**1**	**15**	**positive**	**VP**
**3**	**M**	**76**	**1**	**0**	**0**	**0**	**0**	**2**	**1**	**3**	**3**	**12**	**positive**	**VP**
**4**	**M**	**84**	**1**	**0**	**0**	**0**	**1**	**3**	**2**	**2**	**2**	**13**	**positive**	**VP**
**5**	**F**	**75**	**1**	**1**	**1**	**0**	**0**	**2**	**2**	**2**	**2**	**12**	**positive**	**VP**
**6**	**M**	**84**	**0**	**0**	**0**	**1**	**0**	**3**	**3**	**3**	**3**	**12**	**positive**	**VP**
**7**	**M**	**76**	**0**	**0**	**0**	**0**	**1**	**2**	**2**	**1**	**1**	**15**	**positive**	**VP**
**8**	**F**	**78**	**0**	**0**	**0**	**0**	**1**	**2**	**2**	**2**	**1**	**11**	**positive**	**VP**
**9**	**F**	**78**	**1**	**1**	**0**	**0**	**0**	**4**	**3**	**3**	**3**	**14**	**positive**	**VP**
**10**	**M**	**71**	**0**	**0**	**0**	**0**	**0**	**2**	**2**	**1**	**3**	**14**	**positive**	**VP**
**11**	**M**	**81**	**1**	**0**	**0**	**0**	**0**	**4**	**3**	**2**	**3**	**15**	**positive**	**VP**
**12**	**M**	**68**	**0**	**1**	**0**	**0**	**1**	**2**	**2**	**2**	**2**	**12**	**positive**	**VP**
**13**	**F**	**83**	**1**	**1**	**0**	**0**	**0**	**4**	**3**	**2**	**3**	**10**	**positive**	**VP**
**14**	**F**	**72**	**0**	**0**	**0**	**1**	**0**	**2**	**2**	**1**	**2**	**15**	**positive**	**VP**
**15**	**F**	**79**	**1**	**1**	**0**	**0**	**0**	**2**	**2**	**1**	**2**	**12**	**positive**	**VP**

AD: Alzheimer dementia, CVD: cerebrovascular diseases, GSg,c,u: gait, cognition, urination domains of the grading scale, mRS: modified Rankin scale, LCS: lumbar canal stenosis, Pcsf: cerebrospinal fluid pressure

**Fig 1:**
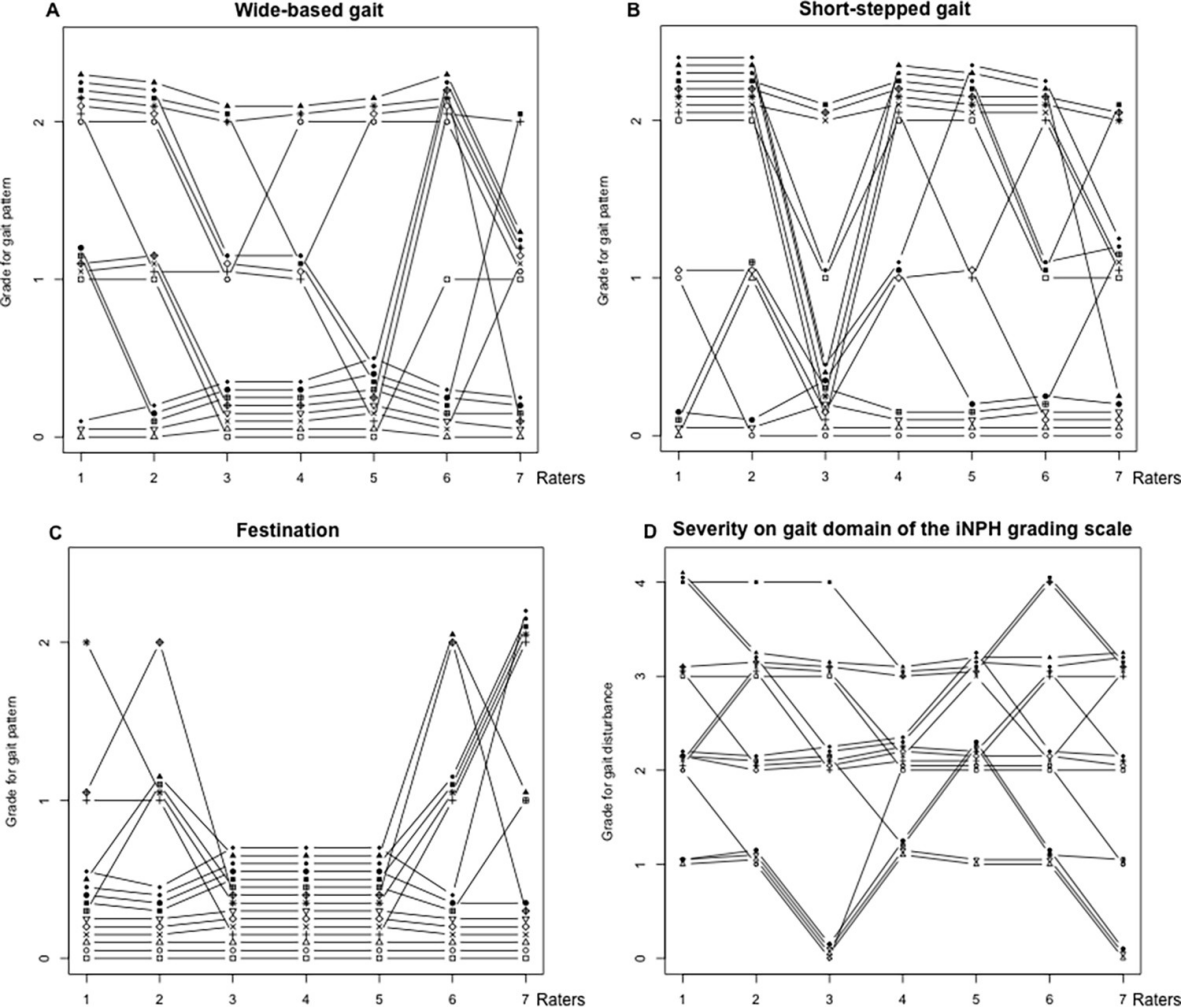
Ratings by seven raters on representative gait patterns and severity of gait disturbance in the first assessment in the homogeneous group. Each grade by different raters was plotted not to overlap. A: Wide-based gait, B: short-stepped gait, C: festination, D: severity on the gait domain of the iNPH grading scale.

The ratings of the seven patterns of gait disturbance and the gait domain of the iNPHGS assessed by three groups of raters in the first and second assessments are shown in S1 Table and S2 Table, respectively. As an example of grades, for wide-based gait in the first session (Fig 1A, S1 Table), raters 1 and 2 (GR2 group) and rater 6 (PTl2 group) showed high incidence on grade 3, whereas raters 3, 4, and 5 (PTe3 group) showed high incidence on grade 0. For short-stepped gait (Fig 1B), rater 3 in the short-stepped gait tended to rate grade 0 compared to other two raters. This reflected low alpha in the PTe3 group. For festination (Fig 1C), all raters of the PTe3 group showed grade 0. This finding was not changed in the second assessment (S2 Table).

The Krippendorff alpha coefficients and their 84% CIs in the five groups (DR2, PTe3, PTI2, PTa5, and all) in the first and second sessions are shown in Table 2. Lack of variation in the PTe3 group for festination yielded alpha = 0, which was considered meaningless. Therefore, festination was excluded from following analyses.

**Table 2:** Krippendorff alpha coefficients and 84% CIs of the five groups.

#	Group	Freezing	Shuffling	Wide-based	Short-stepped	Festination	Standing difficulty	Turning difficulty	GSg
**1**	**DR2(l,u)-1**	**0.42(0.03, 0.72)**	**0.73(0.29, 0.98)**	**0.87(0.71, 0.97)**	**0.94(0.84, 0.99)**	**0.70(0.27, 0.96)**	**0.41(0.18, 0.68)**	**0.64(0.30, 0.88)**	**0.80(0.58, 0.90)**
	**DR2(l,u)-2**	**0.49(0.09, 0.74)**	**0.65(0.31, 0.97)**	**0.18(-0.20, 0.51)**	**0.65(0.35, 0.84)**	**0.55(0.15, 0.77)**	**0.86(0.69, 0.96)**	**0.55(0.16, 0.79)**	**0.90(0.75, 0.98)**
**2**	**PTe3(l,u)-1**	**0.60(0.22, 0.83)**	**0.42(-0.01, 0.69)**	**0.78(0.59 0.91)**	**0.54(0.29, 0.73)**	**0(NA, NA)**	**0.34(0.07, 0.56)**	**0.64(0.30, 0.88)**	**0.69(0.47, 0.80)**
	**PTe3(l,u)-2**	**0.56(0.25, 0.80)**	**0.60(0.31, 0.79)**	**0.74(0.52, 0.91)**	**0.62(0.30, 0.83)**	**0(NA, NA)**	**0.65(0.41, 0.80)**	**0.57(0.15, 0.80)**	**0.65(0.45, 0.76)**
**3**	**PTl2(l,u)-1**	**0.76(0.53, 0.90)**	**0.71(0.41, 0.91)**	**0.41(0.01, 0.70)**	**0.60(0.27, 0.84)**	**0.47(0.12, 0.73)**	**0.24(-0.15, 0.52)**	**0.62(0.23, 0.83)**	**0.87(0.74, 0.92)**
	**PTl2(l,u)-2**	**0.44(0.04, 0.74)**	**0.86(0.64, 1)**	**0.72(0.41, 0.92)**	**0.55(0.16, 0.79)**	**0.29(-0.12, 0.57)**	**0.59(0.22, 0.84)**	**0.87(0.54, 1)**	**0.85(0.64, 0.94)**
**4**	**PTa5 (l,u)-1**	**0.57(0.29, 0.75)**	**0.52(0.31, 0.68)**	**0.62(0.41, 0.75)**	**0.58(0.38, 0.73)**	**0.03(-0.05, 0.10)**	**0.31(0.11, 0.45)**	**0.50(0.22, 0,68)**	**0.74(0.56, 0.80)**
	**PTa5 (l,u)-2**	**0.48(0.24, 0.64)**	**0.63(0.41, 0.77)**	**0.71(0.52, 0.91)**	**0.67(0.37, 0.83)**	**-0.05(-0.12, 0.01) **	**0.62(0.42, 0.75)**	**0.65(0.001, 0.81)**	**0.69(0.49, 0.77)**
**5**	**All7(l,u) -1**	**0.55(0.26, 0.70)**	**0.58(0.36, 0.73)**	**0.62(0.45, 0.73)**	**0.62(0.43, 0.72)**	**0.17(0.06, 0.25)**	**0.40(0.23, 0.54)**	**0.52(0.26, 0.69)**	**0.75(0.59, 0.81)**
	**All7(l,u) -2**	**0.52(0.30, 0.66)**	**0.55(0.32, 0.72)**	**0.58(0.40, 0.69)**	**0.65(0.39, 0.81)**	**0.13(0.03, 0.19)**	**0.62(0.45, 0.75)**	**0.61(0.33, 0.77)**	**0.75(0.59, 0.82)**

Grey cell: Krippendorff alpha ≥0.667, CIs: confidence intervals, DR2: doctor group, l,u: lower and upper limits of CIs, PTe3: experienced physiotherapist group, PTl2: less experienced physiotherapist group

**Fig 2:**
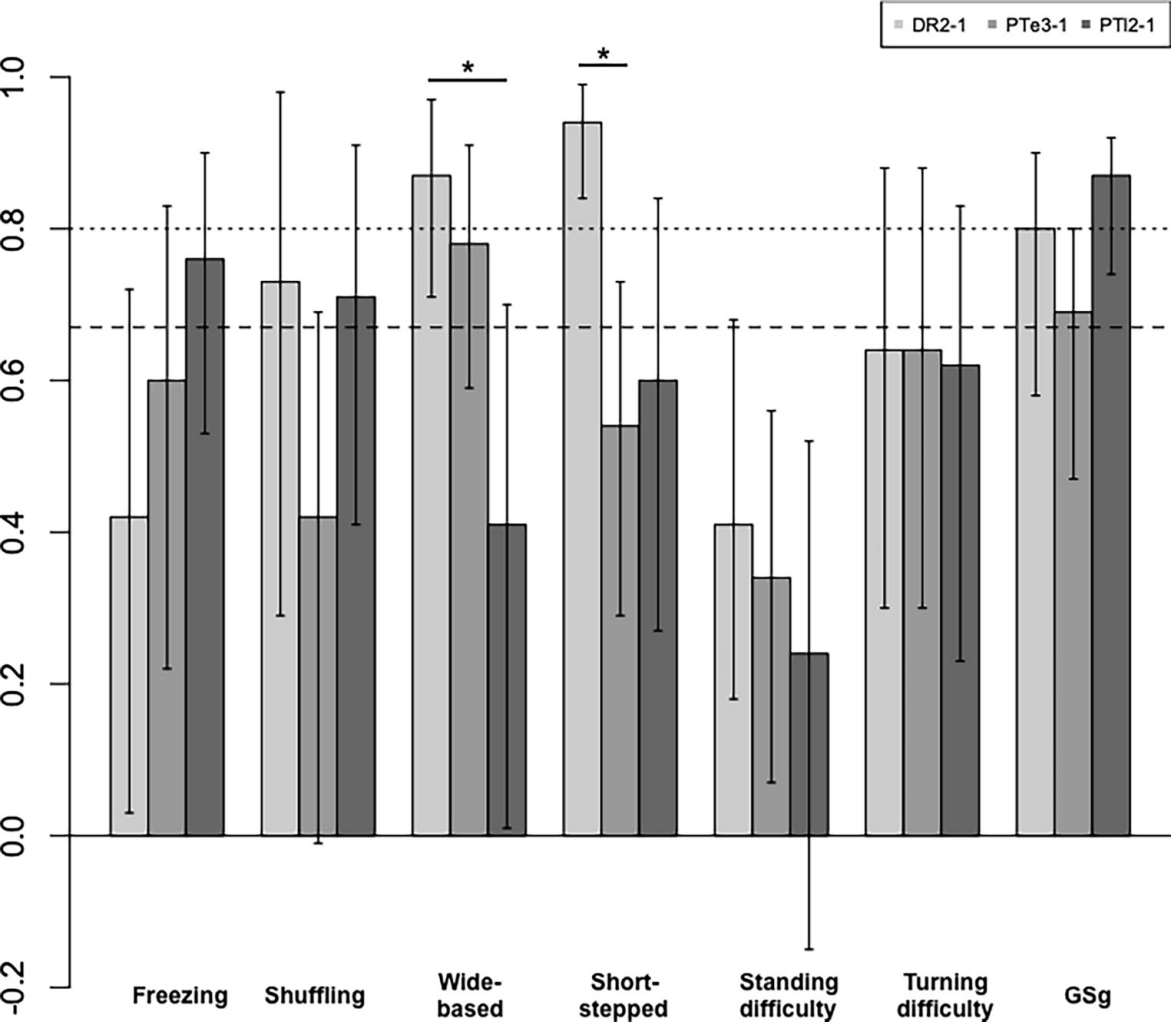
Krippendorff alpha in the first assessment in the homogeneous group. Dashed line: Krippendorff alpha ≥0.667, dotted line: Krippendorff alpha ≥0.80, longitudinal bar: 84% confidence intervals, DR2: doctor group, PTe3: experienced physiotherapist group, PTl2: less experienced physiotherapist group, *statistically significant difference between two groups.

In the first assessment (Table 2, Fig 2), excellent agreement (mean Krippendorff alpha ≥0.0.8) was observed for wide-based gait and short-stepped gait in DR2 among the six gait patterns. There were no gait patterns where two or more groups agreed. Meanwhile, for GSg, excellent agreement was observed by two groups of DR2 and PTl2. Good agreement (alpha ≥0.667) was observed for the following four patterns: freezing gait, shuffling gait, wide-based gait, and short-stepped gait. Good agreement by two groups was observed for the shuffling (DR2 and PTl2) and wide-based (DR2 and PTe3) gaits. For freezing and short-stepped gait, good agreement was observed by only one group. DR2-1 showed good agreement for shuffling, wide-based, and short-stepped gaits. PTe3-1 showed good agreement for wide-based gait. PTl2-1 showed good agreement for freezing and shuffling gaits.

Statistical analysis revealed statistically significant difference (p<0.05) for wide-based and short-stepped gaits in DR2, compared with those of PTl2 or PTe3.

**Fig 3:**
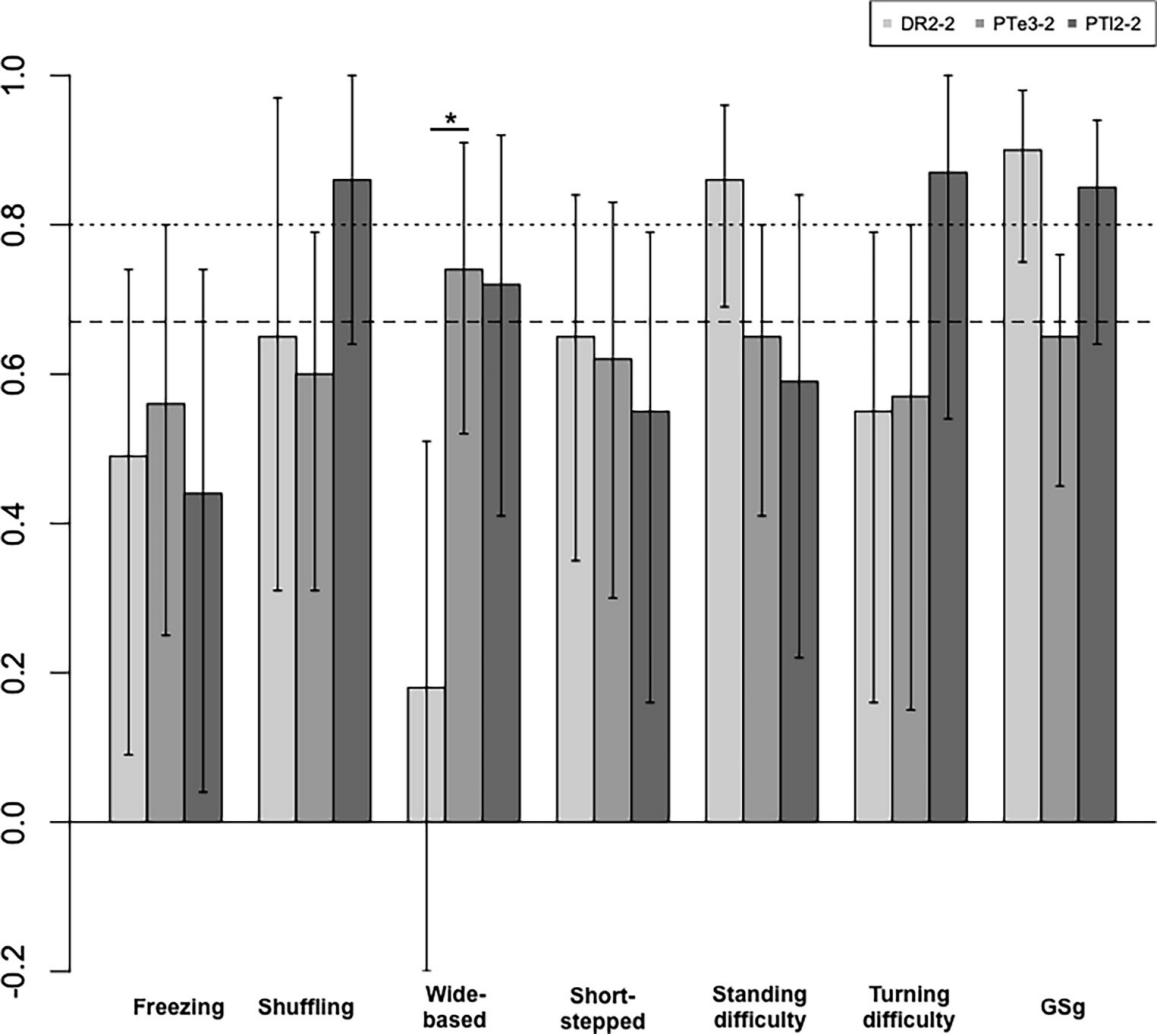
Krippendorff alpha in the second assessment in the homogeneous group. Dashed line: Krippendorff alpha ≥0.667, dotted line: Krippendorff alpha ≥0.80, longitudinal bars: confidence intervals, DR2: doctor group, PTe3: experienced physiotherapist group, PTl2: less experienced physiotherapist group, *statistically significant difference between two groups.

In the second assessment (Table 2, Fig 3), excellent agreement was observed for shuffling, standing difficulty, and turning difficulty. Again, there were no gait patterns where two or more groups agreed. For GSg, excellent agreement was observed by two groups (DR2 and PTl2). Good agreement was observed for shuffling gait, wide-based gait, standing difficulty, and turning difficulty. There were no gait patterns that showed good agreement between two groups or more. Pairwise comparison among the three homogeneous groups showed a statistically significant difference for wide-based gait (p<0.05), with that of DR2-2 being markedly lower than that of PTl2-2.

**Fig 4:**
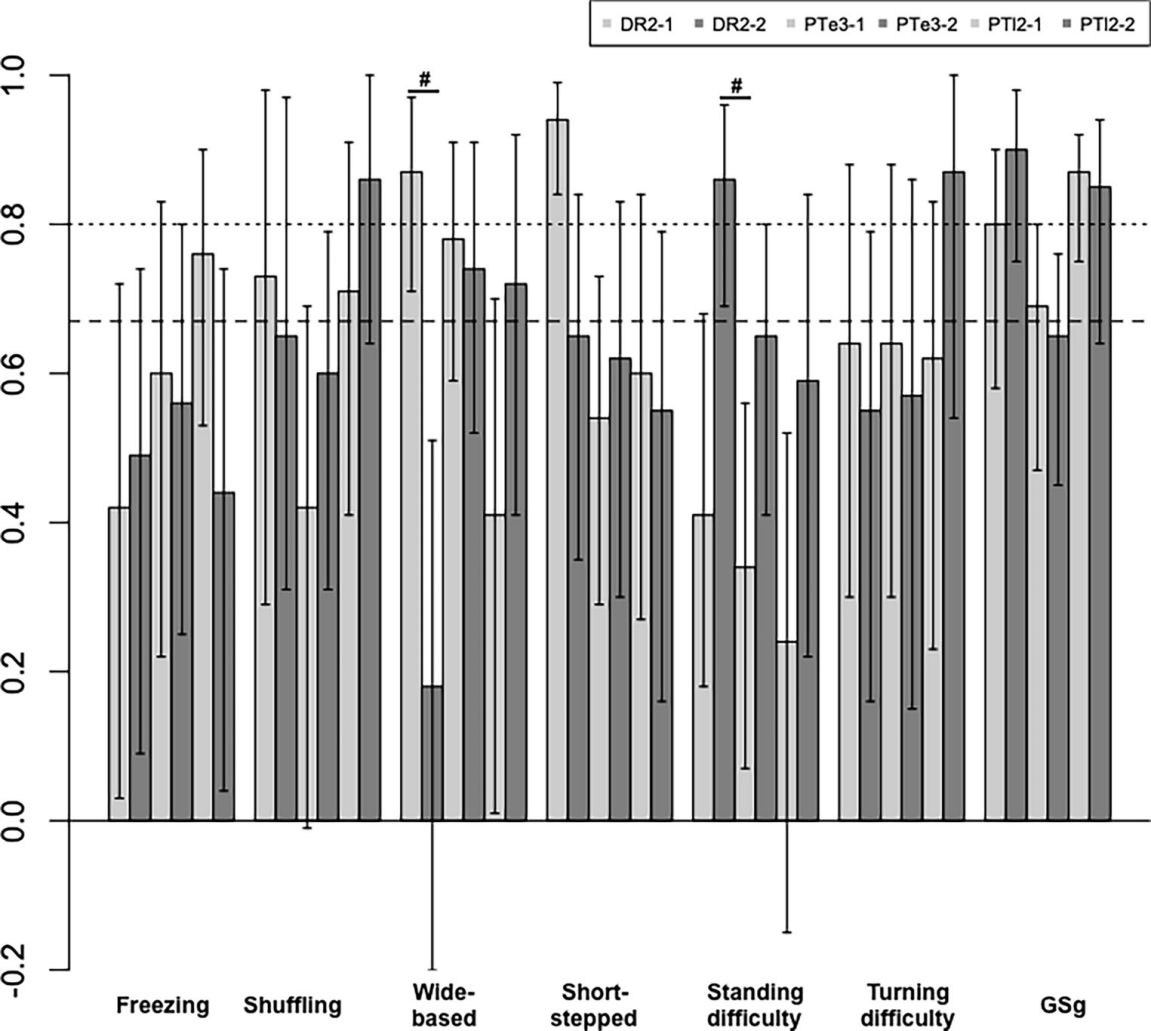
Comparison between the first and second assessments in the homogeneous group. Dashed line: Krippendorff alpha ≥0.667, dotted line: Krippendorff alpha ≥0.80, longitudinal bars: confidential intervals, DR2: doctor group, PTe3: experienced physiotherapist group, PTl2: less experienced physiotherapist group, #statistically significant difference between the first and second assessments.

Comparison between the first and second assessments (Table 2, Fig 4) revealed a statistically significant difference (p<0.05) between wide-based gait and standing difficulty in DR2, with wide-based gait being lower and standing difficulty being higher on the second assessment. Thus, learning session was effective only for standing difficulty in DR2. PTl2-2 showed good agreement for the following three patterns: shuffling gait, wide-based gait, and turning difficulty. These groups showed increased alpha coefficients for the four patterns, but there were no statistically significant differences.

In contrast to the gait patterns, excellent agreement was observed on the GSg for both DR2 and PTl2 groups in both assessments. Good agreement was observed for all three homogeneous groups in the first assessment and for two groups in the second assessment. Even in PTe3-2, the alpha coefficient was 0.65, which was close to a level of good agreement.

**Fig 5:**
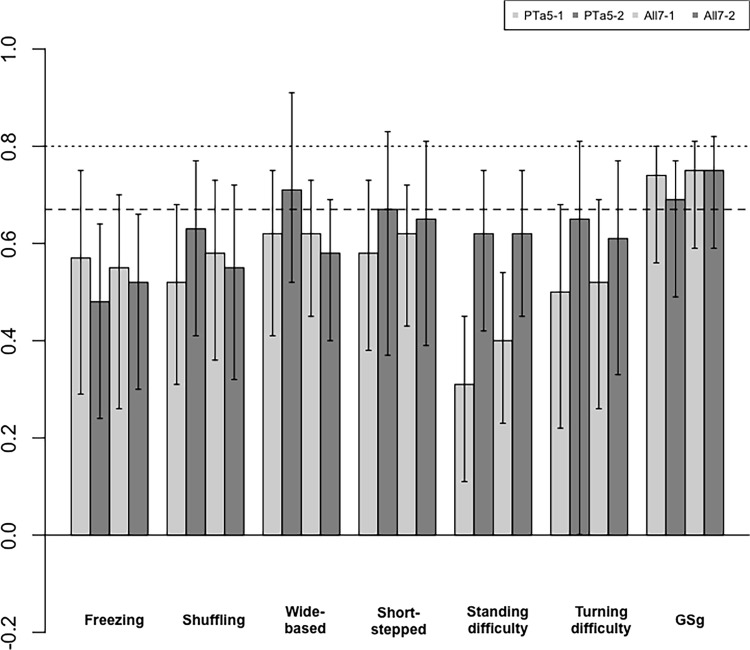
Comparison between the first and second assessments in the mixed group. Dashed line: Krippendorff alpha ≥0.667, dotted line: Krippendorff alpha ≥0.80, longitudinal bars: confidence intervals, DR2: doctor group, PTe3: experienced physiotherapist group, PTl2: less experienced physiotherapist group.

When comparing the first and second assessments between the mixed groups (Table 2, Fig 5), excellent agreement was not observed for any gait patterns and GSg. Good agreement was observed for wide-based and short-stepped gaits in the second assessment, without any statistical significance. Meanwhile, good or almost good agreement was observed again for the GSg, both in the first and second assessments.

## Discussion

This agreement study was performed among DR2, PTe3, and PTl2 groups to examine the gait disturbance in patients with iNPH using a video-assisted rating method. In the first assessment, excellent agreement (alpha ≥0.8) was observed for wide-based gait and short-stepped gait in DR2 among six gait patterns. There were no gait patterns where two or more groups agreed.

Good agreement (alpha ≥0.667) by two groups was observed for the shuffling (DR2 and PTl2) and wide-based (DR2 and PTe3) gaits. The statistical analysis revealed statistically significant difference (p<0.05) for the wide-based and short-stepped gaits in DR2. In the second assessment, there were no gait patterns showing good agreement between two groups or more. Statistically significant difference (p<0.05) among three groups was observed for wide-based gait, with that of DR2-2 being markedly lower than that of PTl2-2. Comparison between the first and second assessments revealed a statistically significant difference in wide-based gait and standing difficulty in DR2, with the former being lower and the latter being higher on the second assessment. Thus, the learning effect was observed only for wide-based gait. In contrast to the gait patterns, excellent agreement on the GSg was observed in the DR2 and PTl2. Good agreement on the GSg was observed for all three homogeneous groups in the first assessment and for two groups in the second assessment. Even in PTe3-2, the alpha coefficient was 0.65, which was close to the level of good agreement. On the GSg for the mixed groups, good agreement was observed again in both the first and second assessments.

At planning of this study, we expected that agreements among three groups for a majority of gait patterns would be the alpha ≥0.667, because alpha <0.667 indicates meaningless. However, this study revealed that the agreement among three groups of different professions or with different degrees of experience was not as good. All of the raters had encountered patients with iNPH in daily practice and provided assessment based on their own experience. The learning effect was not high. These results may be due to the poor expertise of the raters. Better or longer video-assisted lecture may improve the agreement of rater’s assessment. We usually regard the ratings assessed by different physicians or physiotherapists in their respective specialized fields as the same. However, this may not be correct, because comparison of the agreement among multiple raters or groups is rarely performed. Accuracy control is an important issue in hospitals, but it is not regularly performed for subjective ratings among specialists. In clinical practice, there can be variation of grades among multiple raters or multiple groups of raters, but the agreement level should be at least alpha ≥0.667. Below this level, any clinical studies using those measures are not reliable. To confirm the reliability on assessment data, agreement studies should be performed, especially when using qualitative measures such as nominal or categorical scales.

For gait patterns, severity was classified into three grades: none, mild, and evident. This classification is popular and easy for raters, but interrater differences are a matter of concern, as shown in this study. This issue may be overcome through an active learning method [16] or by using recently developed artificial intelligence technologies, which may allow for more reliable assessment of gait patterns [17]. In contrast to the findings of the patterns of gait disturbance, the gait domain of the iNPHGS showed good agreement in all groups. The iNPHGS was first reported by Kubo et al. [11], and many studies of iNPH patients have been performed using this scale [18, 19]. The GSg (gait domain of the iNPHGS) was rated according to the following five grades: normal, complaints of instability, walks without supportive devices, walks with supportive devices, and unable to walk. Descriptions for each grade are easy to understand, allowing for clear differentiations among neighboring grades. This suggests that a precise description facilitating differentiation between neighboring grades is important for good agreement. Thus, the present study using Krippendorff alpha coefficients proved the usefulness of the iNPHGS, especially with regard to gait domain.

Agreement studies are usually performed with two or more raters. However, group comparisons of multiple raters are rare because statistical measures are not available in clinical practice. In this study, we use the Krippendorff alpha coefficient. Agreement studies are usually performed with Cohen kappa coefficient, but this is limited to the agreement between two raters. Fleiss’ kappa can be applied to studies with three or more raters, but Fleiss’ kappa is a generalization of Scott’s Pi, not of Cohen’s kappa, which is limited to nominal data [9]. The Krippendorff alpha coefficient can apply to any number of raters; missing data; any number of values available for coding a variable; binary, nominal, ordinal, and interval data; ratio; and other types; and data of large or even small sample sizes [13]. It enables uniform agreement standards to be applied to data with two or more raters or groups, as performed in the present study.

In this study, confidence intervals were very wide, even with 84% confidence intervals. In some patterns, such as standing difficulty in PTl2 in Fig 2 or wide-based gait in DR2 in Fig 3, lower limits of CIs were negative. The alpha could become negative, in fact, alpha = -1. This results from sampling errors and systematic disagreements—for the former, this occurs when observations are few, each having large effects on the alpha; for the latter, this occurs when observers agree to disagree or pursue opposing interpretations of instructions given to them [13].

The alpha coefficients for festination were low, especially for the PTe3 group, who rated all participants as grade 0 (Table 2, Fig 1C), indicating no variance. This corresponds to Feinstein and Cicchetti’s [20] paradox of “high percent agreement but low kappa.” Krippendorff indicated the importance of variance [14]. He stated that in the absence of variation, researchers would not know whether their measuring instrument can respond to differences among units should they occur. An alpha coefficient of 0 occurs when raters are unable to distinguish among grades of the scale. The experienced physiotherapists in the present study may have considered the TUG Test as unsuitable for assessing festination. It can be observed more clearly when patients walk a long distance.

The present study has several limitations. First, while it only took approximately 15 minutes to obtain consensus on the patterns of gait disturbance in iNPH patients, this consensus-achieving process was not useful for improving the degree of agreement. This may be due to inappropriateness regarding the presented cases that were used for the learning session. The active learning method, such as small group discussion or learning by teaching, was not included in this study [15]. This form of learning may have helped to improve the degree of agreement. Second, the Krippendorff alpha coefficient is not yet commonly used in agreement studies. However, it has the advantage of being widely applicable to multiple raters, missing values, and various types of data. Therefore, it has the potential to be accepted as the gold standard for agreement studies. Third, in the present study, we used an 84% CIs, not 95% CIs, which is also not popular. However, this method is useful for the graphical analyses in agreement studies using the Krippendorff method.

In conclusion, the present study demonstrated that agreement on gait patterns among three groups of raters was not high, but agreement on the iNPHGS was high. This indicates the importance of a precise description to facilitate differentiation between neighboring grades.

## Supporting information

S1 Table Ratings by 7 raters in the first assessment. (XLSX)

S2 Table Ratings by 7 raters in the second assessment. (XLSX)
